# Dual countertraction for extraction of abandoned leads with severe lead‐to‐lead adhesion

**DOI:** 10.1002/joa3.12648

**Published:** 2021-10-22

**Authors:** Masahiro Toba, Toshihiro Nasu, Nobuyoshi Nekomiya, Ryo Itasaka, Takao Makino, Hisashi Yokoshiki

**Affiliations:** ^1^ Department of Cardiovascular Medicine Sapporo City General Hospital Sapporo Japan; ^2^ Division of Medical Engineering Center Sapporo City General Hospital Sapporo Japan

**Keywords:** countertraction, infection, lead adhesion, pacemaker, transvenous lead extraction

## Abstract

Note that firm adhesion between the atrial lead near the proximal electrode and the ventricular lead is present. Simultaneous application of countertraction from the femoral and jugular workstation, i.e. dual countertraction, liberated the two leads from the adhesion.

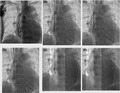

A 92‐year‐old woman with sick sinus syndrome had been implanted with a dual‐chamber pacemaker of two tined leads for 16 years. One month after the second generator replacement, the left pectoral pocket became purulent. The infected pocket was abandoned, leaving two lead fragments in the left subclavian vein, and a new device was implanted (Figure 1A). Five months later, she was referred to our hospital for extraction of the pacemaker leads due to recurrent bacteremia. After the two intact leads on the right were extracted, a Needle’s Eye Snare (94 cm; Cook Medical) was inserted through the femoral workstation for grasping the atrial lead. Maneuver of pulling the proximal end of the atrial lead out of the left subclavian vein was successful. However, lead‐to‐lead binding hampered the release of the two leads despite the right jugular vein approach using another shorter (54 cm) Needle’s Eye Snare for the ventricular lead extraction. The elongated ventricular lead in the jugular workstation was almost completely broken at the binding site (Figure 1B). Therefore, we applied countertraction simultaneously through the both workstations by two operators. This dual countertraction method liberated these leads from the firm adhesion (Figure 1C,D; Movie S1). The long‐drawn‐out ventricular lead was recaptured with the shorter Needle’s Eye Snare at the proximal end, which was pulled up, exposed through the jugular vein and connected to the Bulldog Lead Extender (Cook Medical). Passage of the Byrd dilator sheaths over the extended lead system and application of counterpressure/countertraction facilitated tearing the fibrous tissues near the tricuspid valve, thereby resulted in successful lead extraction (Figure 1E,F).

**FIGURE 1:**
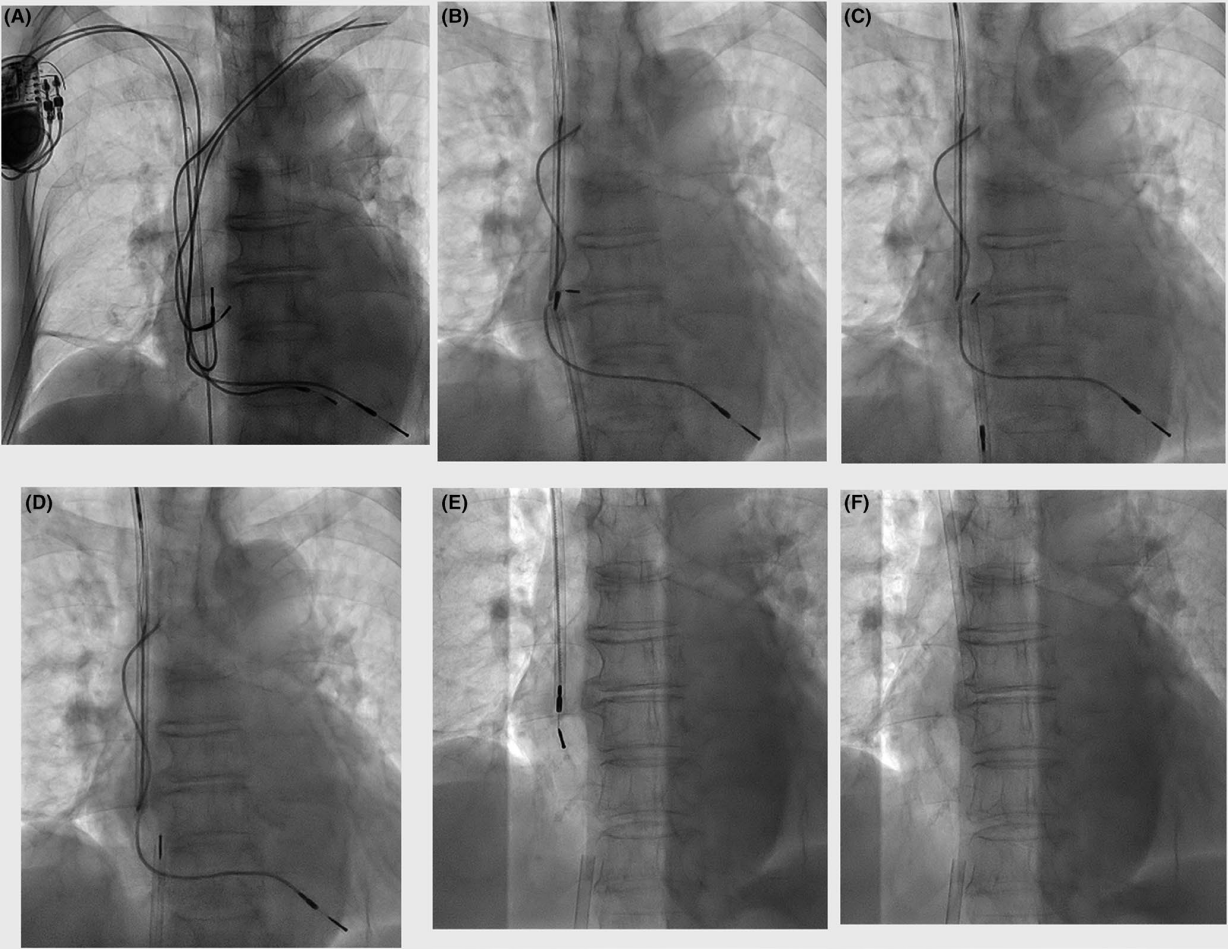
Fluoroscopic images during the procedure: A, Two abandoned leads with proximal endings in the left subclavian vein had been implanted for more than 16 years. A new dual chamber pacemaker was implanted on the right 5 months ago. B, Firm adhesion between the atrial lead near the proximal electrode and the ventricular lead. C, Dual countertraction partially released the adhesion, thereby pulling in the proximal electrode of the atrial lead in the femoral workstation. D, Two leads were completely liberated. E, The ventricular lead was almost extracted; and F, a final image after the lead extraction

Severe lead adhesion is known to be associated with procedural complexity and major complications in percutaneous lead extraction. In this regard, the decision to employ effective alternative approaches appears to be important for successful lead extraction in difficult cases. If we had continued to extract either lead by one operator, the lead must have been broken and teared completely. Because the rate of lead‐to‐lead adhesion was reported to be 18.4% in a total of 936 procedures, the dual countertraction method could be an alternative simple and easy approach especially for extraction of multiple broken leads with proximal‐end migration, which is technically difficult and challenging.

## CONFLICT OF INTEREST

Authors declare no conflict of interests for this article.

## Supporting information

Supplementary Material

